# Health-Seeking Behaviors and its Determinants: A Facility-Based Cross-Sectional Study in the Turkish Republic of Northern Cyprus

**DOI:** 10.15171/ijhpm.2019.106

**Published:** 2019-11-23

**Authors:** Gulifeiya Abuduxike, Özen Aşut, Songül Acar Vaizoğlu, Sanda Cali

**Affiliations:** Medical Faculty, Near East University, Nicosia,TRNC, Turkey.

**Keywords:** Health Seeking Behaviors, Polyclinics, Health Utilization, Determinants, Northern Cyprus

## Abstract

**Background:** Understanding health-seeking behaviors and determining factors help governments to adequately allocate and manage existing health resources. The aim of the study was to examine the health-seeking behaviors of people in using public and private health facilities and to assess the factors that influence healthcare utilization in Northern Cyprus.

**Methods:** A cross-sectional study was conducted in 2 polyclinics among 507 people using a structured intervieweradministered questionnaire. Health-seeking behaviors were measured using four indicators including routine medical check-ups, preferences of healthcare facilities, admission while having health problems, and refusal of health services while ill. Descriptive statistics and multivariable logistic regression analyses were done to explore factors influencing the use of health services.

**Results:** About 77.3% of the participants reported to have visited health centers while they had any health problems. More than half (51.7%) of them had a routine medical check-up during the previous year, while 12.2% of them had refused to seek healthcare when they felt ill during the last five years. Of all, 39.1% of them reported preferring private health services. Current smokers (adjusted odds ratio [AOR]=1.92, 95% CI: 1.17-3.14), having chronic diseases (AOR=2.05, 95% CI: 1.95-2.16), having poor perceptions on health (AOR=2.33; 95% CI: 1.563.48), and spending less on health during the last three months (AOR=2.08, 95% CI: 1.43- 3.01) had about twice the odds of having routine checkups. Higher education (AOR=1.87, 95% CI: 1.38-2.55) was shown to be a positive predictor for the health-seeking behaviors, whereas having self-care problems (AOR=0.18, 95% CI: 0.08-0.40) and having a moderate-income (AOR=0.68, 95% CI: 0.57-0.81) were inversely associated with seeking healthcare.

**Conclusion:** The utilization of public and private health sectors revealed evident disparities in the socio-economic characteristics of participants. The health-seeking behaviors were determined by need factors including chronic disease status and having poor health perception and also by enabling factors such as education, income, insurance status and ability to pay by oneself. These findings highlight the need for further nationwide studies and provide evidence for specific strategies to reduce the socioeconomic inequalities in the use of healthcare services.

## Background

Provision of high-quality health services that are inclusive, affordable, and accessible to all citizens is one of the key responsibilities of the governments around the world.^1^ The World Health Organization (WHO) has set universal health coverage as its number one goal with the tagline of “Health for all - everyone, everywhere” for World Health Day, and governments’ investment in good quality, accessible primary healthcare is the prerequisite of its achievement.^2^ An efficient healthcare utilization, as a result of effective implementation of essential primary healthcare services through universal health coverage, will reduce the disease burden and improve the overall well-being of the population.^3^ Subsequently, it will decrease the economic burden to the country.^1,4-7^

Healthcare utilization is directly related to the healthcare system of the country and health services that are provided.^7,8^ Meanwhile, healthcare utilization and its patterns are reflected by healthcare-seeking behaviors of people. Thus, health services should be planned and provided based on information relating to healthcare-seeking behaviors and utilization, and its determining factors in the context of social, economic, physical, cultural belief, religious practice, gender norms, and political.^9^

Understanding the healthcare-seeking behaviors and its determinants helps government, stakeholders, policy-makers, and health service providers to adequately allocate and manage existing resources, particularly in developing countries.^4,5, 10-12^ Many studies have examined healthcare utilization using Andersen’s behavioral models, where various multidimensional factors determine healthcare-seeking behaviors of people including predisposing factors (age, gender, ethnic, cultural, social), enabling factors (financial, insurance coverage, healthcare accessibility, healthcare availability), and need factors (health perceptions, medical conditions).^6,13-15^

A review by O’Donnell examined healthcare utilization and identified the constraints in accessing healthcare in developing countries. It was summarized that there were vast socioeconomic disparities in the utilizations of healthcare in many low- and middle-income countries. The identified determining factors included income, knowledge, education, cultural and gender roles, social, distance to health facilities, and cost of health services.^4^

The Turkish Republic of Northern Cyprus (TRNC) consists of one-third of the Cyprus island which is home to about 335 000 people with the gross domestic product per capita US$13 897 with a real gross domestic product growth rate of 3.6% in 2016.^16,17^ The population growth rate was 2.0% in 2016, and life expectancy at birth was 84.5 years for females and 80.8 years for males, respectively.^16^

There is no universal health coverage provided in Northern Cyprus, and individuals have several options to seek healthcare services.^18^ One option is the public health facilities provided by the government, which consists of public hospitals and health centers. Health expenditures are greatly reimbursed in public facilities for those who have social security insurance, which is mandatory for everyone in the workforce by the government. The health services in the public sector are provided for free of charge in some emergency situations and accidents, which covers partners and children under 18 years old. However, many people prefer private healthcare facilities such as private hospitals and clinics as they provide better treatment with state-of-the-art advanced health technologies and less waiting time compared to public health facilities.

Being fragmented without a systematic referral system and having high out-of-pocket (OOP) expenditures for healthcare are the main features of the health system in TRNC.^18,19^ In addition, there are no official reports or statistics available on the utilization of health services and health expenditures in the country. This has resulted in an unequal distribution of health facilities and inadequate planning of resources in different regions of the island. In this study, we attempted to fill the information gap by examining the healthcare-seeking behavior of people in public and private health facilities, and also to assess the determining factors that influence the use of healthcare services in Northern Cyprus.

## Methods

### Study Design and Period

A cross-sectional study was conducted in 2 health facilities in Northern Cyprus, namely, Trenyolu polyclinic in Nicosia, and Kyrenia University Dr. Suat Günsel hospital polyclinic in Kyrenia. The study was carried out during October 8-15, 2018.

### Study Area

Northern Cyprus has a growing population, which consisted of mainly Turkish Cypriots, Turkish Republic citizens, and other nationalities. Among the 5 districts in the island, Nicosia is the most populous and Kyrenia is the third with populations of 94 824 and 69 163, respectively.^19^ Based on the registered data in 2015, major causes of mortality were heart diseases, cancers, respiratory diseases, cerebral hemorrhage, hypertension, and internal hemorrhage. Among all deaths, about 60% occurred in Nicosia, while 9% occurred in Kyrenia.^19^

There are a total of 38 health facilities consisting of 24 government sectors and 14 private sectors, including hospitals, polyclinics, and primary care centers. Trenyolu polyclinic is one of the 5 public health facilities located in Nicosia to provide primary healthcare services to the community. The main services include antenatal and neonatal care, child health and women’s health, immunization, dental services, chronic diseases treatment and management, and other integrated health services.

The second study site was a policlinic of the Kyrenia University, Dr. Suat Günsel hospital (which will be referred as Kyrenia polyclinics) which is among the four private health facilities situated in Kyrenia. The private hospital has 150 beds consisting of all health units, including emergency, surgery, cardiology, internal medicine, pediatrics, gynecology and obstetrics, and operating rooms. On the ground floor, there are a number of polyclinics that provide preventive, primary healthcare services such as child and women’s health, chronic diseases control and management, and other services. For this study, only those who came to visit the polyclinics were included.

### Sample Size Estimation and Sampling

A minimum sample size of 383 was calculated using the formula (n = z^2^pq/d^2^) by taking the prevalence (p) of attending healthcare facilities while having illness at 48% with 0.5% precision at 95% confidence level.^20^ A sample size of 421 was used after adding a non-response rate of 10%. To obtain more precise estimation, we recruited more participants (n = 507) for this study. A non-probability convenient sampling method was used, and those aged 15 years and above who attended polyclinics at the time of the study were recruited. A total of 507 individuals (253 and 254 participants from Kyrenia and Trenyolu polyclinics, respectively) participated in the study with a response rate of 84.5%.

The reasons for applying a non-probability sampling method are twofold. Firstly, there is no comprehensive information on primary healthcare centers/polyclinics in Northern Cyprus in order to randomly select the clinics for the study. Secondly, these 2 clinics were relatively bigger in size and provide comprehensive primary care services in the respective regions. The people who visit these clinics include all ethnicities, age groups and nationalities that are similar to the population composition of the island. Moreover, due to the agreement between Near East University and Ministry of Health in terms of medical internship programs, it is relatively easier to obtain official permissions to conduct community surveys in these health facilities.

### Outcome Measurement

The primary outcome of interest in the study was the healthcare-seeking behaviors of respondents. We defined healthcare-seeking behaviors as “the willingness of an individual to seek help when ill and also where a person seeks medical care and preferable treatment among others.”^21^ As a result of an extensive review of literatures, the dependent variable was measured using four indicators that reflect participants’ behaviors in the use of health facilities and preferences. These were routine medical check-ups, admitting to the health centers while having any health problems, refusal of seeking healthcare while feeling sick, and preferences of public and private health centers.

Former three indicators were measured using the questions, “In the past year, have you had any health check-ups without any health complaints at a healthcare center?,” “Do you visit any healthcare centers when you have a health condition?” and “In the past 5 years, have you ever refused to visit a health center, although you were in need of care?” The answers were dichotomized with options of “1 = Yes” and “0 = No.” The latter indicator was measured using a question, “Normally (with the exception of emergency cases) which kind of healthcare centers do you prefer?” with answer options of “1 = Public” and “0 = private.”

### Study Tool

A structured interviewer-administered questionnaire was developed through an extensive literature review and expert validations. A pre-test was conducted through face to face interviews among 30 outpatient visitors of Near East Hospital and excluded from the analysis.

The questionnaire consisted of information about predisposing factors such as age (in years), gender, marital status, number of children, family size, nationality, and education level.^10^ Marital status was categorized into three groups, “married,” “single,” and “others” which included separated, divorced, and widowed. Living conditions and ethnic background play important roles in shaping one’s healthcare seeking behaviors^10,22^ Family size was operationalized as the number of people living in household, where having “four or less (≤4)” people was considered as small (nuclear) families and having “five or more (≥5)” people was classified as having larger families.^23^

The question “How many children do you have?” was asked to know the number of children that the participants have. For the analysis, the answers were coded “1-2,” “3-4,” and “≥5.” To our knowledge, there are a number of foreigners regularly visiting the polyclinic in Kyrenia hospital, mainly British. Thus, nationality was classified as “Turkish,” “Northern Cyprus,” “the UK,” and “Others.” Educational level indicated the highest level of schooling attained, and was classified into three groups, “secondary or less,” “high school,” and “university and above.”

There were questions related to the enabling factors including insurance status, self-rated economic status, OOP expenditure during the last three months, employment status, and types of workplaces. Health insurance status was one of the important factors influencing health-seeking behaviors of people.^11^ Insurance coverage was assessed through a question “How do you pay for your healthcare expenses?” with options as “government insurance,” “private health insurance,” and “own funds.” Self-rated economic status was taken based on respondents’ perception of their own financial status using a question “How do you rate your economic status?”^24^ and answers were categorized as “high,” “moderate,” and “low.”

OOP expenditures consisted of costs spent on medicine, check-ups, diagnostic tests, consultation, and other indirect costs that occurred during the last three months. OOP was assessed by asking “Approximately, how much (Turkish Lira, TL) did you spend during the last three months on your health?” The answers were categorized into three groups “none,” “<500 TL,” “≥500 TL.” Employment status and types of workplaces are directly related to ones’ income and insurance status which determine increased accessibility and preferences of health services.^11,25^ Employment status was assessed by asking if they were currently employed, and the answers were “Yes” and “No.” If the answer was “Yes,” participants were asked if they work in “public sector,” “private sector” or “self-employed,” and for the analysis, the latter 2 were combined as “private sector.” Subsequent questions were regarding the need factors of the participants including self-reported health status, history of having chronic diseases, medicine intake, chronic diseases in the family (Yes/No). Self-reported health status is one of the commonly used predictors of morbidity and mortality among populations.^26^ It was assessed through the question “How do you rate your general health?” with the options “excellent,” “very good,” “good,” “average,” and “poor.” For the analysis, the answers were classified into three groups as “good” (including excellent, very good, and good), “fair,” and “poor.”^26,27^

History of having a chronic disease was assessed with a question “Have you ever been diagnosed by a doctor with one or more chronic illnesses?” with the answer options “Yes” and “No.” The information on medicine intake on a regular basis was obtained through a question “ Do you take any medication on a regular basis?” with answers “Yes” and “No.” Participants’ functional health status was evaluated with a question “Have you ever had any problems with self-care-taking?” and the answers were “Yes” and “No.”

Self-care taking mainly indicated the ability of participants doing the daily activities such as bathing, moving without physical constrains, doing household tasks, using public transport, and doing other activities such as shopping.^24^ Information on smoking status (nonsmoker/current smoker) and alcohol intake (Yes/No) were collected to examine if these risky behaviors are related to the usage of health services.

### Data Analysis

The data were analyzed using IBM SPSS (Statistical Package for the Social Sciences) version 23 (SPSS Inc., Chicago, IL, USA). Descriptive statistics including frequency, percentage, mean, and standard deviation (SD) were done to describe the characteristics of the study sample. Distribution of participants’ socio-demographic characteristics by public and private health centers were analyzed using the chi-square test. Bivariate analysis was done to examine the relationships between socio-demographics, self-reported health status, health facility related factors with 4 outcome indicators that measure participants’ healthcare-seeking behaviors. Exposure variables having *P*  < .05 level of significance in bivariate analysis were entered to construct the final model of multivariable logistic regression analysis. Adjusted odds ratio (AOR) was obtained by clustering variables at the clinical level. AOR and CI were presented with the *P* value set at <.05.

## Results

The mean age of the participants was 42.64 (SD *±* 17.80) years and 53.8% of the participants were women. Table 1 illustrates the distribution of socio-demographic characteristics by health centers which they visited. Most of the characteristics have shown statistically significant relationships with the types of health clinics they visited. Higher proportions of participants with older age, with higher education, better economic status, and who were working in the private sector visited the Kyrenia polyclinic.

**Table 1:** Distribution of Socio-Demographic Characteristics of the Participants by Health Centers (N = 507)

**Socio-Demographic Characteristics**	**Kyrenia Policlinics**		**Trenyolu Policlinics**		**Total**		**χ** ^ 2 ^	***P*** **Value**
	**No.**	**%**	**No.**	**%**	**No.**	**%**		
The subtotal and total number of participants	253	49.9	254	50.1	507	100		
Gender							0.16	.722
Male	119	50.9	115	49.1	234	46.2		
Female	134	49.1	139	50.9	273	53.8		
Age group							13.95	.001^a^
<40	114	45.0	142	56.1	256	50.6		
40-64	89	35.2	89	35.2	178	35.2		
≥65	50	19.8	22	8.7	72	14.2		
Nationality							115.20	.000^a^
TRNC	107	42.3	129	50.8	236	46.5		
Turkish	54	21.3	79	31.1	133	26.3		
British	75	29.6	2	0.8	77	15.2		
TRNC + Turkish	-	-	34	13.4	34	6.7		
TRNC + British	-	-	2	0.8	2	0.4		
Others	17	6.8	8	3.1	25	4.9		
Educational status							46.99	.000^a^
Secondary or less	23	9.1	75	29.5	98	19.3		
High school	63	24.9	80	31.5	143	28.2		
University and above	167	66.0	99	39.0	266	52.5		
Economic status (n = 504)							17.15	.000^a^
Good	118	46.6	88	35.0	206	40.9		
Moderate	128	50.6	135	53.8	263	52.2		
Poor	7	2.8	28	11.2	35	6.9		
Employment							7.68	.006^a^
Public	29	15.3	39	27.9	68	20.7		
Private	160	84.7	101	72.1	261	79.3		
Chorionic diseases							0.17	.704
Yes	83	32.8	79	68.9	162	32.0		
No	170	67.2	175	31.1	345	68.0		
Medicine use							0.06	.844
Yes	73	28.9	70	27.9	143	28.4		
No	180	71.1	181	72.1	361	71.6		
Family size							16.01	.000^a^
≤4	236	93.3	206	81.4	442	87.4		
≥5	17	6.7	47	18.6	64	12.6		
No. of children							20.57	.000^a^
1-2	133	79.6	84	56.4	217	68.7		
3-4	31	18.6	55	36.9	86	27.2		
≥5	3	1.8	10	6.7	13	4.1		
Smoking status							8.05	.005^a^
Non-smoker	181	74.8	158	62.9	339	68.8		
Current smoker	61	25.2	93	37.1	154	31.2		
Alcohol intake							7.99	.005^a^
Yes	63	52.1	72	36.0	135	42.1		
No	58	47.9	128	64.0	186	57.9		
Health expenditure payment methods							92.53	.000^a^
Government insurance	47	23.6	152	76.4	199	39.3		
Private insurance	51	71.8	20	28.2	71	14.1		
Own funds (OOP)	155	65.7	81	34.3	236	46.6		
OOP health expenditure during the last 3 months (TL)							57.94	.000^a^
None	109	43.1	107	42.1	216	42.6		
<500	57	22.5	122	48.0	179	35.3		
≥500	87	34.4	25	9.8	112	22.1		

Abbreviations: TRNC, Turkish Republic of Northern Cyprus; OOP, out-of-pocket; TL, Turkish Lira. ^a^ *P*  < .05.

The proportion of participants with bigger family size and having more than 3 children were significantly higher in Trenyolu polyclinics compared to Kyrenia polyclinics. While the proportion of current smokers (37.1% vs. 25.2%) were significantly higher among Trenyolu visitors, alcohol intake (52.1% vs. 36.0%) was significantly higher among participants who visited Kyrenia polyclinics.

The insurance status of the participants was shown to be one of the statically significant factors as those who had private insurance (71.8% vs. 28.2%) and used own funds (65.7% vs. 34.3%) had chosen Kyrenia polyclinics. Subsequently, the OOP health expenditure ≥500 was significantly higher (34.4% vs. 9.8%) among participants who visited Kyrenia compared to those who visited Trenyolu.

Table 2 presents the relationships between the socio-demographic characteristics of participants with their health-seeking behaviors. Among all, about 77.3% of them said that they had visited health centers while they had any health problems. Participants with higher education level, with higher economic status, and with a better perception of their health had a higher tendency to seek medical services when they had any health problems compared to their counterparts.

**Table 2:** Relationships Between Socio-Demographic Characteristics and Visiting a Health Center in Case of Health Problems, Having a Routine Check-up During the Previous Year, Refusing to Admit a Health Center, and the Frequently Preferred Health Center (N = 507)

**Socio-Demographic Feature**	**Visiting a Health Center in Case of Health Problem**	**Having a Routine Check-up During the Previous Year**	**Refusing to Visit a Health Center Although Feeling Ill During the Last 5 Years**	**Frequently Preferred Health Center**	
				**Government**	**Private**
	**% (n)**	**% (n)**	**% (n)**	**% (n)**	**% (n)**
**Total**	**77.3 (392/507)**	**51.7 (262/507)**	**12.2 (62/507)**	**61.9 (314)**	**38.1(193)**
Gender	ns	a	ns	ns	ns
Male	76.1 (178)	45.7 (107)	10.7 (25)	63.7 (149)	36.3 (85)
Female	78.4 (214)	56.8 (155)	13.6 (37)	60.4 (165)	39.6 (108)
Age group	ns	b	a	ns	ns
<40	78.5 (201)	44.5 (114)	12.1 (31)	62.9 (161)	37.1 (95)
40-64	77.5 (138)	56.7 (101)	15.7 (28)	63.5(113)	36.5 (65)
≥65	72.2 (52)	63.9 (46)	4.2 (3)	55.6 (40)	44.4 (32)
Nationality	ns	ns	c	c	c
TRNC	79.6 (215)	54.4 (147)	11.1(30)	67.0 (181)	33.0 (89)
Turkish	72.9 (97)	45.1 (60)	21.1 (28)	75.2 (100)	24.8 (33)
British and Others	76.9 (80)	52.9 (55)	3.8 (4)	31.7 (33)	68.3 (71)
Educational status	b	ns	c	c	c
Secondary or less	65.3 (64)	55.1 (54)	22.4 (22)	78.6 (77)	21.4 (21)
High school	75.5 (108)	50.3 (72)	15.4 (22)	67.8 (97)	32.2 (46)
University and above	82.7 (220)	51.1 (136)	6.8 (18)	52.6 (140)	47.4 (126)
Employment status	ns	b	ns	ns	ns
Yes	83.8 (57)	45.0 (117)	12.3 (32)	59.2 (154)	40.8 (106)
No	78.2 (204)	58.8 (144)	12.2 (30)	64.5 (158)	35.5 (87)
Economic status	a	ns	a	b	b
High	83.0 (171)	53.9 (111)	8.3 (17)	54.4 (112)	45.6 (94)
Moderate	74.5 (196)	49.0 (129)	13.3 (35)	65.0 (171)	35.0 (92)
Low	62.9 (22)	57.1 (20)	22.9 (8)	80.0 (28)	20.0 (7)
Smoking status	ns	c	ns	a	a
Current smoker	78.2 (265)	58.4 (198)	10.6 (36)	59.0 (200)	41.0 (139)
Non-smoker	76.0 (117)	37.7 (58)	16.2 (25)	70.1 (108)	29.9 (46)
Chronic disease	ns	c	a	ns	ns
Yes	72.8 (118)	67.3 (109)	16.7 (27)	58.6 (95)	41.4 (67)
No	79.4 (274)	44.3 (153)	10.1 (35)	63.5 (219)	36.5 (126)
Medication use	ns	c	ns	ns	ns
Yes	74.1 (106)	65.0 (93)	15.4 (22)	61.5 (88)	38.5 (55)
No	78.7 (284)	46.5 (168)	10.8 (39)	61.8 (223)	38.2 (138)
Perceived health status	b	c	a	ns	ns
Good	82.1 (161)	41.8 (82)	8.7 (17)	59.2 (116)	40.8 (80)
Fair	77.9 (173)	51.8 (115)	12.2 (27)	64.0 (142)	36.0 (80)
Poor	65.2 (58)	73.0 (65)	20.2 (18)	62.9 (56)	37.1 (33)

Abbreviation: TRNC, Turkish Republic of Northern Cyprus. ns: non-significant (*P*  > .05); ^a^*P*  < .05, ^b^*P*  < .01; ^c^*P*  < .001.

More than half (51.7%) of the participants reported that they had done routine medical checkups during the previous year. Females and older participants tend to do routine checkups more frequently compared to males and the younger age group. Participants who were smokers, who had chronic diseases, and those who were taking medications, and those who had poor health perceptions tend to go for medical checkups more frequently compared to their counterparts.

Among all, 12.2% of the participants had refused to seek healthcare during the last 5 years even though they felt ill. Participants with a lower educational level, who were Turkish Republic citizens, and those with lower economic status had a higher tendency of refusing to seek healthcare compared to others.

About 62.0% of the participants reported preferring government health facilities, while the rest (38.1%) mentioned that they prefer the private sector. Participants who were Turkish nationals, with lower economic status and low education level tend to prefer public sector more compared to the private sector.

The relationship between health-facility related factors and health-seeking behaviors were presented in Table 3. More than 82% of participants who visited Kyrenia reported to seek healthcare if they are sick, and the majority (61.7%) also mentioned to prefer the private health sector over the public health sector.

**Table 3:** Health Facilities Related Characteristics of Those Who Had Visited a Health Center in Case of Health Problems, Who Had Routine Check-up During the Previous Year, Who Had Refused to Visit a Health Center, and the Frequently Preferred Health Center (N = 507)

**Health Facility Related Factors and Others Features**	**Visiting a Health Center in Case of Health Problem**	**Having a Routine Check-up During the Previous Year**	**Refusing to Visit a Health Center Although Feeling Ill During the Last 5 Years**	**Frequently Preferred Health Center**	
				**Government**	**Private**
	**% (n)**	**% (n)**	**% (n)**	**% (n)**	**% (n)**
Health center	b	ns	c	c	c
Kyrenia	82.2 (208)	51.4 (130)	5.5 (14)	38.3 (97)	61.7 (156)
Trenyolu	72.4 (184)	52.0 (132)	18.9 (48)	85.4 (217)	14.6 (37)
Health insurance	ns	ns	ns	c	c
Government	77.4 (154)	53.8 (107)	15.1 (30)	79.4 (158)	20.6 (41)
No insurance (OOP)	77.1 (182)	49.6 (117)	10.6 (25)	51.3 (121)	48.7 (115)
Private	77.5 (55)	52.1 (37)	9.9 (7)	49.3 (35)	50.7 (36)
The money spent for health for the last 3 months (TL)	ns	c	ns	c	c
None	77.3 (167)	40.7 (88)	10.6 (23)	65.7 (142)	34.3 (74)
<500	74.9 (134)	60.9 (109)	13.4 (24)	74.3 (133)	25.7 (46)
≥500	81.3 (91)	58.0 (65)	13.4 (15)	34.8 (39)	65.2 (73)
No. of household members	b	ns	a	a	a
≤4	79.4 (351)	53.2 (235)	10.9 (48)	60.0 (265)	40.0 (177)
≥5	62.5 (40)	40.6 (26)	21.9 (14)	75.0 (48)	25.0 (16)
Self-care problem	b,d	ns	ns	ns	ns
Yes	38.5 (5)	46.2 (6)	23.1 (3)	61.5 (8)	38.5 (5)
No	78.3 (387)	51.8 (256)	11.9 (59)	61.9 (306)	38.1 (155)

Abbreviations: TRNC, Turkish Republic of Northern Cyprus; OOP, out-of-pocket; TL, Turkish Lira. ns: non-significant (*P*  > .05); ^a^*P*  < .05; ^b^*P*  < .01; ^c^*P*  < .001; ^d^ Fisher exact test.

The proportion of participants who refused to seek healthcare was significantly higher in Trenyolu polyclinics compared to Kyrenia polyclinics. Health insurance status of the participants was shown to have a statistically significant relationship with their preferences of public and private health centers. Those who had government insurance preferred to go to government health center or hospital compared to those who did not.

Participants who went for routine checkups and also those who reported to prefer the private sector tend to spend more (≥500 TL) on health compared to those who did not. The proportion of participants with larger family size (≥5 people) have a significantly higher tendency to seek healthcare when they have any health problems and prefer public sector compared to their counterparts.

The results of multivariable logistic regression analysis were presented in Table 4. Factors such as having high education, moderate economic status, and having self-care problems were significantly attributed to participants’ decision on seeking health services if they have any health problems. Participants who had university and above education level (AOR = 1.87, 95% CI: 1.38-2.55) were nearly twice as likely to seek healthcare compared with those of lower education level. Individuals with self-care problems (AOR = 0.18, 95% CI: 0.08-0.40) and those with moderate income (AOR = 0.68, 95% CI: 0.57-0.81) were less likely to seek health services as compared to those who did not. Going for routine medical checkups during the previous year was significantly associated with being a current smoker, having chronic diseases, having poor health perception, and having the ability to spend less than 500 TL for health services.

**Table 4:** Factors Associated With Visiting to a Health Center in Case of Health Problems and Doing a Routine Check-up During the Previous Year – Logistic Regression Analysis

**Factors**	**Visiting a Health Center in Case of Health Problems**		**Doing a Routine Check-up During the Previous Year**	
	**AOR**	**95% CI**	**AOR**	**95% CI**
Gender (male vs. female)			0.76	(0.45-1.29)
Age group				
<40			Ref	
40-64			1.20	(0.82-1.77)
≥65			1.35	(0.79-2.31)
Educational status				
Secondary or less	Ref			
High school	1.42	(0.68-2.95)		
University and above	1.87	(1.38-2.55)^a^		
Employment status (Yes vs. No)			0.83	(0.76-0.90)^a^
Economic status				
High	Ref			
Moderate	0.68	(0.57-0.81)^a^		
Low	0.72	(0.28-1.83)		
Smoking status (Smoker vs. non-smoker)			1.92	(1.17-3.14)^a^
Chronic disease (Yes vs. No)			2.05	(1.95-2.16)^a^
Medication use (Yes vs. No)			0.68	(0.55-0.84)^a^
Health Perception				
Good	Ref		Ref	
Fair	0.92	(0.65-1.29)	1.01	(0.66-1.56)
Poor	0.60	(0.35-1.01)	2.33	(1.56-3.48)^a^
The money spent on health for the last 3 months (TL)				
None			Ref	
<500			2.08	(1.43-3.01)^a^
≥500			1.63	(0.78-3.42)
No of household members (≤4 vs. ≥5)	1.99	(0.63-6.25)		
Self-care problem (Yes vs. No)	0.18	(0.08-0.40)^a^		

Abbreviations: AOR, adjusted Odds Ratio; TL, Turkish Lira. ^a^ *P*  < .001.

Current smokers had 1.92 (95% CI: 1.17-3.14) times the odds of doing routine checkups, while those who were having chronic disease (AOR = 2.05, 95% CI: 1.95-2.16), having poor perceptions (AOR = 2.33; 95% CI: 1.56-3.48), and having the ability to spend less than 500 TL for health services during the last 3 months (AOR = 2.08, 95% CI: 1.43-3.01) were twice as likely to do routine checkups compared to those who did not. Moreover, those who were employed (AOR = 0.83; 95% CI: 0.76- 0.90), and those who were taking medication regularly (AOR = 0.68; 95% CI: 0.55- 0.84) were shown to have fewer medical checkups compared to those who did not.

## Discussion

To the best of our knowledge, this study is the first of its kind to provide a snapshot regarding the health-seeking behaviors, patterns of healthcare utilization and its determining factors in Northern Cyprus. Information on the behaviors of people in seeking healthcare services are essential to form evidence-based health policies and efficiently manage the resources in the country based on the demand and influencing factors.^4,28-30^ Health seeking behaviors are determined by physical, political, socioeconomic, cultural factors, gender norms, and health system.^4,5,11,12,31^

In the present study, 61.9% of the participants said that they prefer public health center rather than the private sector. This might be because most of the participants were covered by government insurance and the cost of healthcare is more affordable in Trenyolu compared to Kyrenia hospital. Apart from some characteristics including gender, chronic condition, and medicine use, most of the socioeconomic and demographic factors were demonstrated to have significant effects on the preferences in seeking healthcare in public or private health facilities.

The majority of the participants from the United Kingdom and other countries had chosen to seek health services in private facilities, while most of the TRNC and the Turkish citizens had preferred public health facilities. The main reason for foreigners choosing Kyrenia polyclinics might be due to the better English language proficiency of the doctors and close distance to where they live. Most importantly, this trend might be also due to the lack of a systematic primary healthcare system in Northern Cyprus, which leads to easy access to specialists and general practitioners in tertiary hospitals without any referral from primary care physicians.

The preference of private sector also positively correlated with participants’ higher educational level, higher income, and employment in the private sector. Moreover, the proportion of respondents who had paid more than 500 TL for health during the last three months were significantly higher among those who visited the private sector. The findings were consistent with the study results by Masiye and Kaonga conducted in Zambia, which had reported that socioeconomic factors such as household economic capacity, educational level, employment status of the head of the household influence the choice of health facilities and the amount of OOP expenditure.^32^

In the current study, higher income and educational level contributed to the higher OOP expenditure, were evidently demonstrated as the significant determinants of the health-seeking behaviors, which was comparable to the results from other studies.^4,22,28,30,32-36^ In this study, insurance status of the participants was shown to be one of the significant determinants in choosing the types of health facilities, which is in line with several studies conducted in Ireland,^37^ Pakistan,^35^ Ghana,^22^ Malaysia,^11^ and China.^38^ However, it was not a significant determinant in shaping the health-seeking behaviors of our study participants in terms of the other three indicators.

This result was contrary to the results from a study by Mou et al, which reported that insured migrant workers were more likely to visit doctors and use public health facilities when they are sick compared to the uninsured workers in Shenzhen, China.^38^ Similar findings were reported by Abu Bakar and Samsudin, which highlighted that the insurance coverage was the significant factor in the private healthcare utilization in Malaysia, while not as significant in the usage of the public health sector.^11^

Out of 507 participants, 51.7% of them had gone through routine medical checkups without any health complaints during the last year, while 12.2% of them had refused to seek healthcare even if they felt sick during the last 5 years. Meanwhile, 77.3% of the participants reported that they have had seek healthcare when they had any health problem. The results of the bivariate analysis have shown that being females, older age, and being employed, current smokers, having chronic diseases, medication use, and poor perception of self-health status were the characteristics of the participants that significantly influenced their health-seeking behaviors. This is consistent with studies from Canada,^31^ Ethiopia,^39^ Kenya,^30^ Vietnam,^40^ China,^41^ Korea,^14^ Turkey,^13^ and Malaysia.^11^ For instance, Thompson and colleagues had examined the cross-sectional Patient Experiences Survey collected from 7260 patients in ten Canadian provinces and had found that women have visited primary healthcare physicians in response to physical and mental health issues to a greater extent compared to men. Also, other factors were shown to have significant influences on a patient’s health-seeking behaviors, such as age, income, and having chronic conditions.^6,31,41^

Unlike the other studies,^11,14,39^ marital status was not a significant determinant for healthcare utilization in our study. The results of the logistic regression analysis of our data did not show statistical significances for predisposing factors such as gender, age, family size, however, enabling factors such as being smokers, having middle income status, having chronic diseases, higher educational level, having poor health perception, and moderate payment for health services (<500 TL) were demonstrated to be significant determinants for health-seeking behaviors of our participants.

Contrasting results were seen in a study from Korea by Kim and Lee, which has found no significant effects of enabling factors including educational level, economic, and insurance status on healthcare utilizations among outpatients.^14^ This might be due to the country’s healthcare system and services provided. In our study, the main reasons for medical check-ups were prescribing medicines, routine follow-ups, and treatment of respiratory and other chronic diseases. Perceived severity of self-rated health condition was shown to be one of the main determinants for seeking healthcare along with having chronic diseases. This result was supported by the previous studies reporting that need factors are the strongest determinants of the healthcare utilization.^6,13,14,29,36,41^

Several factors were shown to be statistically significant determinants for visiting a healthcare facility when they had any health problems, including having university and above education level, medium income status, and having self-care problems. Highly educated individuals were nearly 2 times as likely to use healthcare services, whilst those who had self-care problems and moderate-income level had a significantly lower probability of using healthcare. The main reason for this pattern might be as for those who have self-care problems most probably were dependent on the care of their family members, and most of the time they might have severe health conditions that need specialized care in the hospital settings.

The results were contrary to the findings from a study by Sözmen and Ünal, which examined inequalities in healthcare utilization among Turkish adults. The study highlighted that income inequality shaped the pattern of healthcare utilization in Turkey, and individuals with lower education, lower income, living in rural areas have a higher tendency to visit primary care physicians, while those who have bigger household size (≥5) had a lower probability to visit primary care physicians compared to their counterparts.^13^ These factors were similar to the characteristics of the participants in our study related to the refusal of seeking healthcare even if they were sick during the last 5 years, which were having a lower education level, lower income, being Turkish nationals, having a poor self-rated health condition, and having the larger household.

There are several limitations to the study. First, the cross-sectional study design with convenient sampling methods might limit the reliability and generalizability of the study results to the entire population, and the causal relationships between factors should be interpreted with caution. A non-probability sampling method was used as there is a lack of comprehensive information on all health facilities in order to randomly select representative study sites. Hence, we aimed to take a snapshot of the health-seeking behaviors of people who visited the selected 2 health centers during the time of the study. Second, data was collected using a structured interviewer-administered questionnaire which relied on self-reports by the participants. Most probably it might lead to information and recall bias. Particularly, due to incomplete information regarding chronic conditions, medicine use, and self-perceived health status, which might lead to underestimation of the effects of these factors. Third, health-seeking behaviors was measured using 4 outcome indicators, as it is impossible to obtain information about the objective measurements such as the total number of outpatient and inpatient visits, health expenses on each visit including types of treatment and costs. However, one of the strengths of the current study is to provide evidence on health-seeking behaviors, healthcare utilization, and determining factors, which sets a foundation for the further rigorous studies in this area. Nationwide comprehensive studies should be carried out systematically by the government to obtain information on the current health services provided, health needs, health expenditures, and health-seeking behaviors of the population to develop an efficient primary healthcare system in the country as it is an essential part of the comprehensive healthcare system. A number of steps should be taken and specific strategies should be implemented to transform the entire healthcare system of the country, which will eventually benefit the country’s economy and overall well-being of the population.

## Conclusion

The study results revealed evident disparities in socio-demographic characteristics of people who utilized public and private health facilities. Health-seeking behaviors of the participants was significantly related to the factors such as income level, education, age, insurance status, chronic illness, self-perceived health status, ability to pay for OOP, and family size. Moreover, need factors such as having chronic diseases, having self-care problems, and having poor health perceptions along with other predisposing factors were the strongest determinates of the healthcare utilization patterns. The study findings highlighted the needs of systematic collection of information on the usage of health services and health seeking behaviors of people. This would help policy-makers and stakeholders setting up specific strategies to ensure the effective utilization and distribution of existing resources, and to enforce the sufficient delivery of healthcare services in the country.

## Acknowledgements

The study was a part of the intern project of sixth year medical students in Near East University, Nicosia, TRNC, Turkey. Authors would like to thank all the intern students and study participants for their contributions to this study. Authors also would like to thank the management in Kyrenia and Trenyolu polyclinics for their approval and support during the study.

## Ethical issues

The official permission was taken from the health centers to conduct the study. The written consent was obtained and information about the study was provided to the participants prior to the data collection. The questionnaires were anonymous and all procedures of the study were in accordance with the Helsinki Declaration ethical standards, as revised in 2000. The manuscript has been read and approved by all researchers. All authors have contributed to the study in accordance with the guides of the Council of Science Editors (CSE) and the International Committee of Medical Journal Editors (ICMJE). The ethical approval from Near East University, Nicosia, TRNC, Turkey was not required for the study as the data were collected using a survey questionnaire.

## Competing interests

Authors declare that they have no competing interests.

## Authors’ contributions

GA was involved in the study design, data collection, data entry, analysis, and drafted the original manuscript. SAV was involved in the study design, data collection, data entry and data analysis. OA and SC contributed to the study design particularly the questionnaire development, data collection, and data entry. All authors read and approved the final manuscript.

## Key messages

**Implications for policy makers**

- Evident disparities in the socioeconomic status of the participants who used public and private health facilities have indicated that people with higher socioeconomic status tend to prefer and utilize private healthcare services compared to their counterparts.
- Factors such as income level, education, age, insurance status, ability to pay for health services, and family size have influenced the healthseeking behaviors of people. Moreover, need factors such as having chronic diseases and having poor health perception along with other predisposing factors were the strongest determinates of the usage of healthcare services.
- Evidence highlighted the need in building a comprehensive and inclusive primary healthcare service with a strong referral system in the country, which is one of the root solutions to reduce the inequalities in healthcare utilization among communities.
- Further comprehensive studies on healthcare utilization and its influencing factors by the government is needed to fill the gap in knowledge.
- Based on the evidence, policy-makers and stakeholders should set up specific strategies to ensure the adequate distribution of existing resources and to enforce sufficient delivery of healthcare services in the country.

**Implications for the public**

Utilization of healthcare services reflects health-seeking behaviors of people, which is determined by physical, social, political, economic, and cultural factors as well as the health system of the country. The present study findings revealed significant socioeconomic disparities among people who visited the public and private health sector. The use of healthcare services was strongly determined by need factors include having chronic diseases and having poor health perception along with various enabling and predisposing factors. The results highlighted the importance of systematically gathering data on health-seeking behaviors and determining factors in order to build a comprehensive primary healthcare system under the universal health coverage for all population.
